# Biosynthetic ability of diverse basidiomycetous yeast strains to produce the natural antioxidant ergothioneine

**DOI:** 10.1186/s13568-024-01672-w

**Published:** 2024-02-09

**Authors:** Shun Sato, Azusa Saika, Kazunori Ushimaru, Tatsuyuki Koshiyama, Yukihiro Higashiyama, Tokuma Fukuoka, Tomotake Morita

**Affiliations:** 1 Research Institute for Sustainable Chemistry, National Institute of Advanced Industrial Science and Technology (AIST), Central 5-2, 1-1-1 Higashi, Tsukuba, Ibaraki 305-8565 Japan; 2 Research and Development Division, Kureha Corporation, 16, Ochiai, Nishiki-Machi, Iwaki, Fukushima 974-8686 Japan

**Keywords:** Ergothioneine, Basidiomycetous yeast, *Pseudozyma*, *Ustilago*, Antioxidant

## Abstract

Sixteen strains of basidiomycetous yeasts were evaluated for their capability to produce ergothioneine (EGT), an amino acid derivative with strong antioxidant activity. The cells were cultured in either two synthetic media or yeast mold (YM) medium for 72 h, after which cytosolic constituents were extracted from the cells with hot water. After analyzing the extracts via liquid chromatography-mass spectrometry (LC-MS), we found that all strains produced varying amounts of EGT. The EGT-producing strains, including *Ustilago siamensis*, *Anthracocystis floculossa*, *Tridiomyces crassus*, *Ustilago shanxiensis*, and *Moesziomyces antarcticus*, were subjected to flask cultivation in YM medium. *U*. *siamensis* CBS9960 produced the highest amount of EGT at 49.5 ± 7.0 mg/L after 120 h, followed by *T*. *crassus* at 30.9 ± 1.8 mg/L. *U*. *siamensis* was also cultured in a jar fermenter and produced slightly higher amounts of EGT than under flask cultivation. The effects of culture conditions, particularly the addition of precursor amino acids, on EGT production by the selected strains were also evaluated. *U*. *siamensis* showed a 1.5-fold increase in EGT production with the addition of histidine, while *U*. *shanxiensis* experienced a 1.8-fold increase in EGT production with the addition of methionine. These results suggest that basidiomycetous yeasts could serve an abundant source for natural EGT producers.

**Supplementary Information:**

The online version contains supplementary material available at 10.1186/s13568-024-01672-w.

## Keypoints

Various basidiomycetous yeasts produced ergothioneine (EGT).

*Ustilago siamensis* possesses great capability of producing EGT.

Precursor amino acids promoted EGT production by *U. siamensis* and *U. shanxiensis*.

## Introduction

**Scheme 1:**
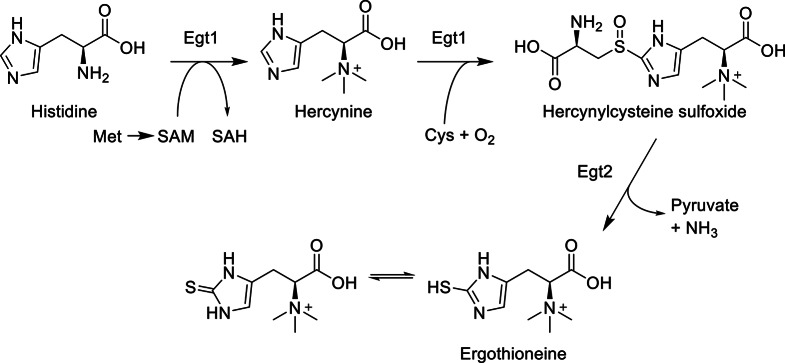
Ergothioneine biosynthetic pathway in fungi. SAM, *S*-adenosylmethionine; SAH, *S*-adenosyl-l-homocysteine

Ergothioneine (EGT) is a naturally occurring l-histidine derivative, containing a betaine structure and a thiol group attached to an imidazole ring (see the chemical structure shown in Scheme 1). Its thiol-thione tautomerism and unique standard redox potential make it a highly stable antioxidant (Cheah and Halliwell 2012; Servillo et al. 2015; Borodina et al. 2020). EGT can scavenge free radicals and reactive oxygen species (Kimura et al. 2005; Stoffels et al. 2017) and reduce oxidative damage in mammals (Colognato et al. 2006; D’Onofrio et al. 2016). Furthermore, there is potential for EGT to prevent or treat neurodegenerative and cardiovascular diseases (Yang et al. 2012; Smith et al. 2020). These properties have led to increased academic interest in EGT, as well as its application in food and pharmaceutical industries, with dietary supplements and cosmetic products containing EGT already being commercialized (Fu and Shen 2022).

The first identified native EGT producer was the ergot fungus *Claviceps purpurea*, followed by various mycobacteria, cyanobacteria, ascomycetes, and basidiomycetes (Cheah and Halliwell 2012; Fu and Shen 2022). Mushrooms, many of which belongs to *Basidiomycota*, are the most popular EGT producers (Lin et al. 2015; Kalaras et al. 2015). However, due to the long cultivation time and low EGT content of mushroom fruiting bodies, alternative, safe, and cost-effective industrial processes are needed to meet the growing demand for EGT. Recent studies have demonstrated EGT production with less cultivation time by mycelial cultivation rather than fruiting bodies of mushrooms such as *Lentinus edodes*, *Pleurotus eryngii*, *Pleurotus citrinopileatus*, and *Panus conchatus* (Tepwong et al. 2012; Liang et al. 2013, 2015; Zhu et al. 2022). Another developing technique for the efficient production of EGT is to create novel EGT producers with genetic engineering technology. Model microorganisms such as *Escherichia coli* and *Saccharomyces cerevisiae* incapable of producing EGT originally have been genetically engineered to produce EGT for potential methods of industrial EGT production (Tanaka et al. 2019; van der Hoek et al. 2019; Hoek et al. 2022a, b). These studies demonstrated gram-scale EGT production by genetically modified microorganisms.

On the contrary, native EGT producers are yet to be fully explored for developing applications of EGT. Fujitani et al. (2018) investigated EGT production by methylotrophic bacteria as well as yeasts and fungi from NBRC RD strains and plants. They found that yeast strains producing EGT included *Rhodotorula mucilaginosa* and strains of *Pseudozyma* and *Sporobolomyces* species belonging to the phylum *Basidiomycota*. Strains of the subphylum *Ustilaginomycotina*, such as *Ustilago* and *Pseudozyma* strains, produce large amounts of glycolipid-type biosurfactants, such as mannosylerythritol lipids and cellobiose lipids (Morita et al. 2015). Our research group has been investigating the use of various basidiomycetous yeast strains to produce glycolipid-type biosurfactants as functional, sustainable materials (Morita et al. 2010, 2011, 2014; Saika et al. 2017, 2020), and many *Pseudozyma* strains, some of which inhabit phyllosphere, were identified as glycolipid producers. However, the production of other functional chemicals, including EGT, by these strains has not fully investigated. In this study, we screened for native EGT producers among various basidiomycetous yeast strains that have been investigated for the production of glycolipids. Multiple strains were found to produce EGT inside cells, and we examined cultivation conditions for selected strains to improve EGT production.

## Materials and methods

### Strains, media, and culture condition

The yeast strains used in this study are listed in Table 1 and were obtained from the Japan Collection of Microorganisms (JCM; RIKEN, Ibaraki, Japan), the Centraalbureau voor Schimmelculues (CBS; The Westerdijk Institute, Utrecht, The Netherlands), the Biological Resource Center (NBRC; National Institute of Technology and Evaluation, Chiba, Japan) and laboratory isolates (Konishi et al. 2007; Morita et al. 2010, 2011). Sixteen strains were screened for EGT production in test tube cultures using mineral medium (MM) (Alamgir et al. 2015; Fujitani et al. 2018); mineral salt (MS) medium composed of 50 g/L glucose, 3 g/L NaNO_3_, 1 g/L yeast extract, 0.3 g/L KH_2_PO_4_, and 0.3 g/L MgSO_4_·7H_2_O; and yeast mold (YM) medium (Becton Dickinson and Co., Franklin Lakes, NJ, USA). Stock cells of the strains were first cultured on a YM agar plate at 25 °C, after which cells grown on the plates were transferred to test tubes containing respective media and shaken at 200 rpm and 25 °C.

**Table 1:** Preliminary examination of EGT-producing basidiomycetous yeast strains in test tube^*a*^

Strains	EGT production (mg/L-culture) in		
	MM medium	MS medium	YM medium
*Anthracocystis flocculosa* JCM10321^T *b*^	1.8	2.3	27.3
*Dirkmeia churashimaensis* OK96	3.2	1.4	14.4
*Kalmanozyma fusiformata* JCM3931^T^	1.3	trace ^*c*^	1.7
*Moesziomyces antarcticus* JCM10317^T^	1.4	1.4	5.8
*Moesziomyces aphidis* JCM10318^T^	1.3	trace	trace
*Moesziomyces parantarcticus* JCM11752^T^	1.9	4.1	19.4
*Moesziomyces rugulosus* NBRC10877^T^	2.1	1.9	13.2
*Pseudozyma alboarmeniaca* CBS9961^T^	trace	1.4	18.2
*Pseudozyma graminicola* CBS10092^T^	1.3	1.2	13.6
*Pseudozyma hubeiensis* KM59	4.6	2.9	20.1
*Pseudozyma prolifica* JCM10319^T^	1.3	trace	trace
*Pseudozyma tsukubaensis* 1E5	2.7	1.1	10.9
*Triodiomyces crassus* CBS9959^T^	2.0	5.8	25.2
*Ustilago maydis* UM521^T^	1.3	1.0	9.1
*Ustilago shanxiensis* CBS10075^T^	1.7	1.8	20.0
*Ustilago siamensis* CBS9960^T^	1.7	5.0	40.1

^a^Cells were cultivated in test tubes containing 3 mL of respective media at 200 rpm and 25 °C for 72 h (*n* = 1)

^b^T, type strain

^c^Trace, less than 1.0 mg/L

To further evaluate EGT production, flask cultivation was performed in 300 mL Erlenmeyer flasks containing 50 mL YM medium, which were inoculated with 0.5 mL test tube YM medium cultures after 2 days of cultivation. The flasks were shaken at 25 °C and 200 rpm for 120 h. When necessary, filter-sterilized amino acid solution was added to the flasks at the beginning of the cultivation.

For jar fermenter experiments, cells of *U. siamensis* were cultured in two 50 mL of YM medium for 2 days, followed by inoculated into 2 L of YM medium in 5 L jar fermenter Bioneer·N-5 L (B. E. Marubishi Co. LTD., Tokyo, Japan). Operation of jar fermenter cultivation was conducted as follows: stirring speed at 400 rpm, cultivation temperature at 25 °C, air flow at 2 L/min (1 vvm). Aliquot of cultures were taken from the jar at appropriate point to analyze optical density at 600 nm (OD_600_) and EGT production inside cells. After 120 h of operation, cells were harvested by centrifugation and dry cell weight were determined.

### Analytical procedures

After cultivation, cells from approximately 1 mL culture were harvested by centrifugation at 4,000 × g for 5 min to remove the culture media and washed once with deionized water. Obtained cell pellets were disrupted in 0.5 mL deionized water and heated at 96 °C for 10 min to extract cytosolic metabolites, including EGT. After cooling to room temperature, cell debris was removed by centrifugation, and the supernatant was diluted and subjected to liquid chromatography-mass spectrometry (LC-MS) analysis. The effluents were separated with a Shodex Asahipak NH2P-40 2D column (Shodex, Tokyo, Japan) and a NH2P-50G guard column (Shodex) at 40 °C that were connected to a Shimadzu LCMS-2020 system (Shimadzu, Kyoto, Japan) with a photodiodearray detector and an electrospray-ionization mass spectrometer (ESI-MS). A 30/70 (v/v) mixture of 10 mM ammonium formate and acetonitrile was used as the mobile phase at 0.1 mL/min. An ion mass spectrum (+) of 230.1 m/z was used to quantify EGT in the extracts. Authentic EGT (Cayman Chemicals, Ann Arbor, MI, USA) was used to construct a calibration curve.

Cell growth was determined using the OD_600_ of the culture medium or dry cell weight. The OD_600_ was measured using a UV-Vis spectrophotometer (V-630, JASCO, Tokyo, Japan) after diluting the culture medium. Dry cell weight was measured by centrifuging the culture medium to harvest cells, followed by washing cells once with deionized water to remove the culture media. The obtained cell pellets were lyophilized to obtain dried cells and weighed to quantify dry cell weight.

## Results

### EGT production of basidiomycetous yeast strains

**Fig. 1:**
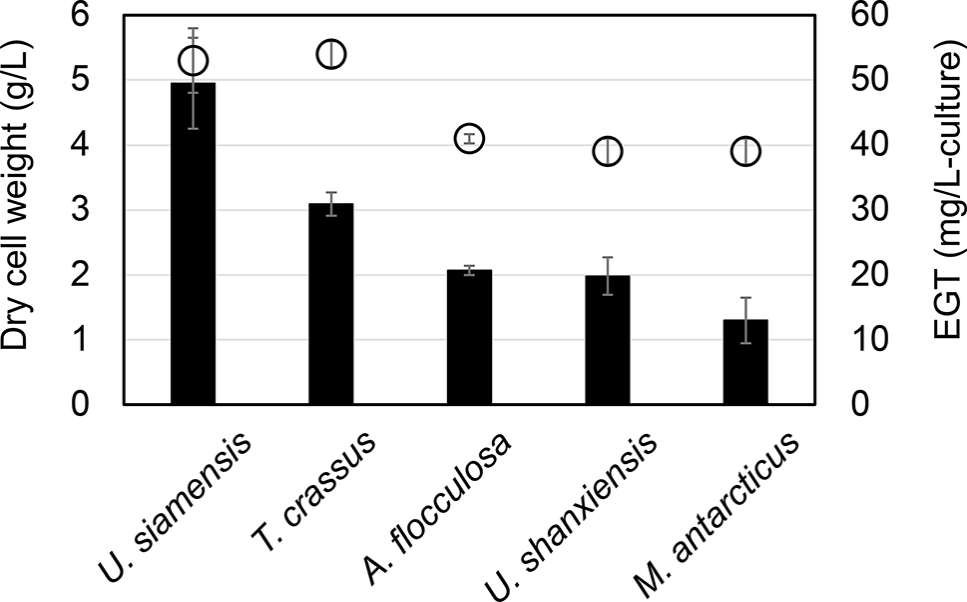
EGT production by selected basidiomycetous yeast strains in flask cultivation. Cells were cultivated in YM medium at 25ºC for 120 h. Data are means and standard deviations from at least three replicates. Circles, dry cell weight; solid bars, EGT

We first examined basidiomycetous yeast strains from culture collections for their ability to produce EGT in test tube cultures using three types of media. As shown in Table 1, MM medium containing glucose as a carbon source yielded only small amounts of EGT (1.3–4.6 mg/L) after 72 h cultivation. The same trend was observed in MS medium, which also contains glucose, with the highest amount of EGT (5.8 mg/L) produced by *Triodiomyces crassus* CBS9959. By contrast, many strains cultivated in YM medium increased their EGT production, with *Ustilago siamensis* CBS9960 producing the highest amount (40.1 mg/L), followed by *Anthracocystis flocculosa* (27.3 mg/L) and *T*. *crassus* (25.1 mg/L). Then we selected five strains for further examination of EGT biosynthesis in basidiomycetous yeasts, specifically three strains with high EGT production (*U*. *siamensis* CBS9960, *T*. *crassus* CBS9959, and *A*. *flocculosa* JCM10321), one strain with moderate EGT production (*U*. *shanxiensis* CBS10075), and one strain with low EGT production (*Moesziomyces antarcticus* JCM10317). These strains were cultivated in flasks containing YM medium, and their EGT production was evaluated (Fig. 1). *U*. *siamensis* CBS9960 and *T*. *crassus* CBS9959 produced EGT at 49.5 ± 7.0 and 30.9 ± 1.8 mg/L, respectively. *A*. *flocculosa* JCM10321 and *U*. *shanxiensis* CBS10075 produced EGT at around 20 mg/L, followed by *M*. *antarcticus* JCM10317 producing less EGT.

### EGT production of *U*. *Siamensis* in a jar fermenter

**Fig. 2:**
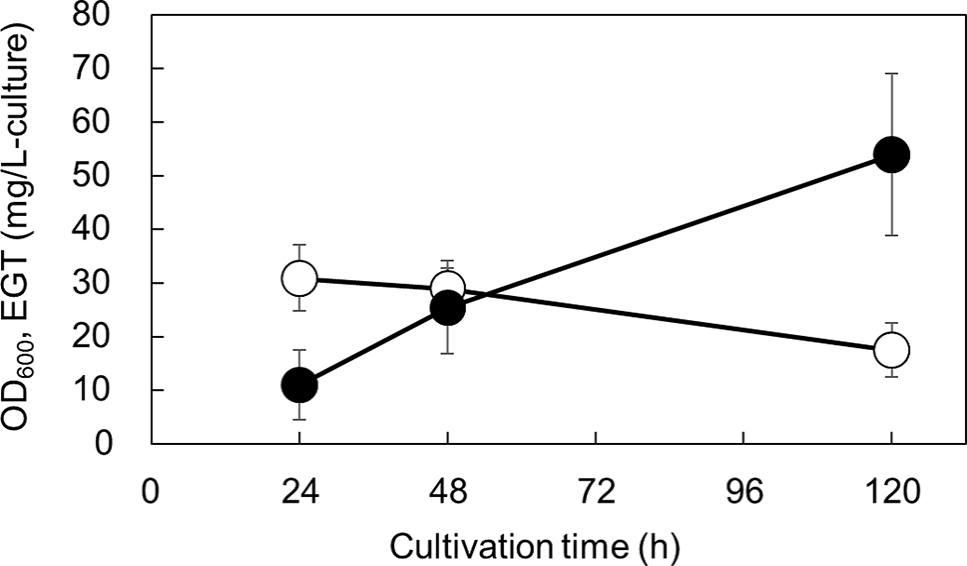
Time-dependent changes of OD_600_ and EGT production by *U. siamensis* using 5 L jar fermenter. Cells were cultivated in YM medium for 120 h (*n* = 11). Open circles, OD_600_; closed circles, EGT

To further investigate the EGT production of *U*. *siamensis* CBS9960, the greatest EGT producer in the flask cultivation experiment, jar fermenter experiments were conducted. Using YM medium, *U*. *siamensis* grew within 24 h of cultivation, followed by a decrease in cell density (Fig. 2). EGT production only began after cell growth and was maintained with further culture time. At 120 h of cultivation, *U*. *siamensis* produced EGT at a titer of 54.0 ± 15.0 mg/L, which was slightly higher than that obtained from flask cultivation (see Fig. 1). This showed that *U*. *siamensis* can produce EGT on a larger scale, and further optimization of culture conditions is necessary to increase EGT productivity by this strain.

### Effect of culture conditions for EGT production

To increase EGT production, the culture conditions, including medium components, temperature, aeration, salinity, and initial pH, were preliminarily investigated using three strains of *U*. *siamensis* (a high-level EGT producer), *U*. *shanxiensis* (a moderate-level EGT producer), and *M*. *antarcticus* (a low-level EGT producer). Increasing glucose concentration in YM medium did not result in an increase in EGT production by these strains (Supplemental Fig. S1), although the dry cell weight of all strains increased to 13.4–15.6 g/L. Addition of yeast extract, an amino acid source in YM medium, slightly increased EGT production in *U*. *siamensis*, although the other two strains showed a decrease in EGT production. Peptone, another amino acid source in YM medium, did not have a positive effect on EGT production. Other culture conditions, including cultivation temperature, aeration, salinity, and initial pH did not result in an increase in EGT production.

**Fig. 3:**
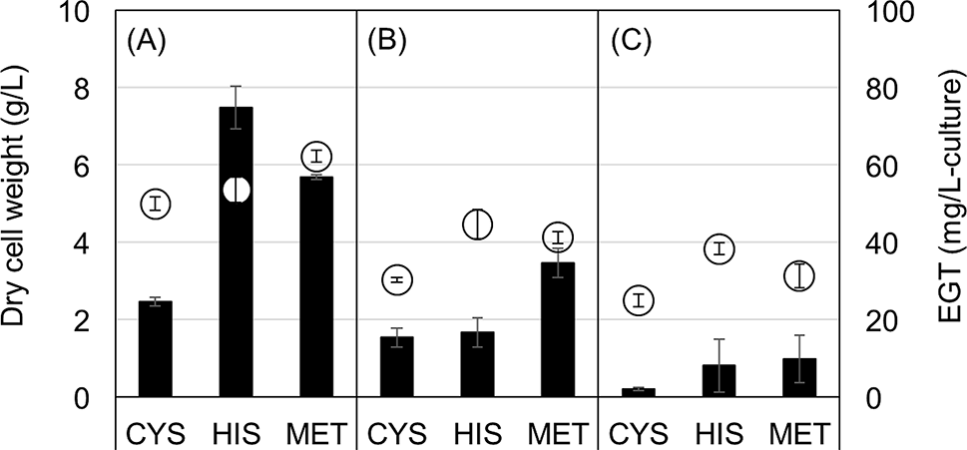
Effect of precursor amino acids on EGT production in (**A**) *U. siamensis*, (**B**) *U. shanxiensis*, and (**C**) *M. antarcticus*. Cells were cultivated in YM medium plus 1 g/L cysteine (CYS), histidine (HIS), or methionine (MET) for 120 h. Circles, dry cell weight; solid bars, EGT. Data are means and standard deviations from three replicates

The amino acids cysteine, histidine, and methionine are the precursors in the EGT biosynthetic pathway (Pluskal et al. 2014; van der Hoek et al. 2022a; see Scheme 1) and thus adding yeast extract, an amino acid source in YM medium, could be promising to increase EGT production in *U*. *siamensis*. To determine the effect of precursors on EGT production in these strains, we evaluated adding precursor amino acids at 1 g/L to YM medium. All strains showed a decrease in dry cell weight and EGT production after adding 1 g/L cysteine (Fig. 3), likely caused by the toxic effect of external cysteine on the cells. After adding histidine, *U*. *siamensis* produced 1.5 times more EGT, whereas *M*. *antarcticus* and *U*. *shanxiensis* did not show a change in EGT production. By contrast, after adding methionine, *U*. *shanxiensis* produced 1.8 times more EGT, while the growth and EGT production of *M*. *antarcticus* and *U*. *siamensis* was unchanged (Fig. 3). These results suggest that histidine and methionine, precursor amino acids for EGT biosynthesis, promoted EGT production in *Ustilago* strains while the availability of precursor amino acids varied among the species.

## Discussion

We found that various ustilaginomycetous yeast strains belonging to *Basidiomycota* were able to produce EGT. Production was greater in YM medium than in MM medium or MS medium. This is likely due to the fact that EGT is a histidine derivative, and its biosynthesis requires cysteine and *S*-adenosylmethionine (Pluskal et al. 2014; van der Hoek et al. 2022a; see Scheme 1). YM medium, which contains an abundance of amino acid sources such as peptone and yeast extract, is likely to support EGT production of ustilaginomycetous yeast strains.

Among the strains tested, *U*. *siamensis* was the highest EGT producer, producing 49.5 mg/L EGT in flask cultivation. *U*. *siamensis* showed a greater level of EGT production in culture medium, intracellular content, and productivity than other native EGT producers, except for mycelia cultivation of *Pleurotus citrinopileatus* and *Panus conchatus* under optimized conditions (Table 2). The production level of EGT by *U. siamensis* was further increased to 75 mg/L by boosting the precursor histidine, with the greatest EGT productivity (13.9 mg-EGT/g-dry cell) among native EGT producers. Because other ustilaginomycetous strains, such as *M*. *antarcticus* and *P*. *tsukubaensis*, have already been used for chemical production in commercial settings (Kitamoto 2019), ustilaginomycetous yeasts may be a viable candidate for large-scale production of EGT.

**Table 2:** EGT production by native producers

Microbial strains	Culture media	EGT (mg/L-culture)	EGT (mg/g-dry cell)	EGT (mg/L/d)	References
*Ustilago siamensis* CBS9960	YM medium (flask)	49.5 ± 7.0	9.3	9.9	This study
	YM medium (jar)	54.0 ± 15.0	11.7	10.8	This study
	YM medium, 0.1% histidine (flask)	74.9 ± 5.5	13.9	15.0	This study
*Tridiomyces crassus* CBS9959	YM medium (flask)	30.9 ± 1.8	5.7	6.2	This study
*Ustilago shanxiensis* CBS10075	YM medium, 0.1% methionine (flask)	34.7 ± 3.9	8.4	6.9	This study
*Oscillatoria* sp. CCAC M1944	Waris-H medium	-	0.8–0.9	-	Pfeiffer et al. (2011)
*Methylobacterium aquaticum* 22 A	MM medium, 2% methanol	12.2	2.0	1.7	Fujitani et al. (2018)
*Nocardia asteroides*	Wickerham medium, 1% mannitol + 0.4% asparagine	-	0.52	-	Genghof (1970)
*Streptomyces griseus* ATCC10317	RN medium	-	0.5	-	Genghof (1970)
*Shizossacharomyces pombe* WT 972	EMM2 medium, nitrogen starvation	-	157.4 (µM, intracellular)	-	Pluskal et al. (2014)
*Rhodotorula mucilaginosa* z41c	SD medium, 2% glycerol + 2% yeast extract	24	3.2	3.4	Fujitani et al. (2018)
*Aureobasidium pullulans* kz25	SD medium, 2% glycerol + 2% yeast extract	14	1.0	2	Fujitani et al. (2018)
*Aspergillus oryzae* NSAR1	Steeped rice solid medium	11.5 (mg/kg-media)	-	2.3	Takusagawa et al. (2019)
*Pleurotus citrinopileatus* (mycelia)	Basal medium, 2% glucose	18.2	2.9	0.83	Lin et al. (2015)
	Basal medium, 2% glucose + amino acids	98	12.3	6.1	Lin et al. (2015)
*Panus conchatus* (mycelia)	Optimized fermentation medium, 5% molasses, 3% soypeptone	81.44	8.4	20.4	Zhu et al. (2022)
	Optimized fermentation medium, 5% molasses, 3% soypeptone, 0.04% cysteine	148.79	9.1	24.8	Zhu et al. (2022)

Recently, gram-scale production of EGT by submerged fed-batch cultivation was achieved through genetic modification of non-EGT producers. Recombinant *E*. *coli*, *S*. *cerevisiae*, and *Yarrowia lipolytica* expressing EGT biosynthetic genes from native EGT producers were able to biosynthesize EGT at 1.31 g/L in 216 h, 2.39 g/L in 160 h, and 1.63 g/L in 220 h, respectively (Tanaka et al. 2019; van der Hoek et al. 2022a, b). Supplementing precursor amino acids to the culture media supported high-level EGT production at 5.4 g/L in 96 h by fed-batch cultivation of the recombinant *E. coli* strain expressing mutants of EGT biosynthetic genes (Zhang et al. 2023). In addition, *Corynebacterium glutamicum*, a popular amino acid producer, was engineered for EGT production by introducing EGT biosynthetic genes and enhancing precursor amino acid biosynthesis, yielding 264.4 mg/L EGT (Kim et al. 2022). Genetic engineering technology for *Ustilaginomycetes* has already been developed for *U*. *maydis* and *M*. *anarcticus* (Olicon-Hernandez et al. 2019; Saika et al. 2017, 2020), and the genomes of multiple ustilaginomycetous strains have been analyzed to date (Kämper et al. 2006; Morita et al. 2014; Wada et al. 2021). Thus, the EGT-producing strains found in this study could also be improved to produce more EGT by introducing heterologous genes for EGT biosynthetic pathways and related metabolic reactions, as well as using self-cloning strategies to boost gene expression for EGT biosynthesis. Our trials of genetically modified ustilaginomycetous strains for enhanced EGT production are currently underway. It should be noted that native EGT producers may have greater tolerance and accumulation capacity than non-EGT producers due to their constant intercellular exposure to EGT. This property may be suitable for concentrating EGT from culture broths by collecting cells, depending on the downstream processes for EGT production.

To date, many biochemical and pharmacological applications of EGT have been explored (Cheah and Halliwell 2012; Borodina 2020). Although genetically modified microorganisms have demonstrated the production of large amounts of EGT, non-genetically modified microorganisms may be more suitable for food ingredients, cosmetic products, and toiletry products. In addition, the physiological role of EGT in native EGT producers has yet to be elucidated. The variety of basidiomycetous yeast strains capable of producing EGT could lead to the development of an industrial EGT production process by scaling-up fermentation and creating genetically modified strains, as well as to a better understanding of microbial EGT production and its physiological roles.

### Electronic supplementary material

Below is the link to the electronic supplementary material.

**Supplementary Material 1: Fig. S1:** Effect of culture conditions on EGT production by (A) *U. siamensis*, (B) *U. shanxiensis*, and (C) *M. antarcticus*

## Data Availability

Not applicable.

## References

[CR1] Alamgir KM, Masuda S, Fujitani Y, Fukuda F, Tani A (2015). Production of ergothioneine by *Methylobacterium* species. Front Microbiol.

[CR2] Borodina I, Kenny LC, McCarthy CM, Paramasivan K, Pretorius E, Roberts TJ, van der Hoek SA, Kell DB (2020). The biology of ergothioneine, an antioxidant nutraceutical. Nutr Res Rev.

[CR3] Cheah IK, Halliwell B (2012). Ergothioneine: antioxidant potential, physiological function and role in disease. Biochim Biophys Acta.

[CR4] Colognato R, Laurenza I, Fontana I, Coppede F, Siciliano G, Coecke S, Aruoma OI, Benzi L, Migliore L (2006). Modulation of hydrogen peroxide-induced DNA damage, MAPLs activation and cell death in PC12 by ergothioneine. Clin Nut.

[CR5] D’Onofrio N, Servillo L, Giovane A, Casale R, Vitiello M, Marfella R, Paolisso G, Balestrieri ML (2016). Ergothioneine oxidation in the protection against high-glucose induced endothelial senescence: involvement of SIRT1 and SIRT6. Free Rad Biol Med.

[CR6] Fu T-T, Shen L (2022). Ergothioneine as a natural antioxidant against oxidative stress-related diseases. Front Pharmacol.

[CR7] Fujitani Y, Almagir KM, Tani A (2018). Ergothioneine production using *Methylobacterium* species, yeast, and fungi. J Biosci Bioeng.

[CR8] Gengho DS (1970). Biosynthesis of ergothioneine and hercynine by fungi and *Actinomycetales*. J Bacteriol.

[CR12] Kalaras MD, Richie JP, Calcagnotto A, Beelman R (2015). Mushrooms: a rich source of the antioxidants ergothioneine and glutathione. Food Chem.

[CR13] Kämper J, Kahmann R, Bölker M, Ma L-J, Brefort T, Saville BJ, Banuett F, Kronstad JW, Gold SE, Müller O, Perlin MH, Wösten HAB, de Vries R, Ruiz-Herrera J, Reynaga-Peña CG, Snetselaar K, McCann M, Pérez-Martín J, Feldbrügge M, Basse CW, Steinberg G, Ibeas JI, Holloman W, Guzman P, Farman M, Stajich JE, Sentandreu R, González-Prieto JM, Kennell JC, Molina L, Schirawski J, Mendoza-Mendoza A, Greilinger D, Münch K, Rössel N, Scherer M, Vraneš M, Ladendorf O, Vincon V, Fuchs U, Sandrock B, Meng S, Ho ECH, Cahill MJ, Boyce KJ, Klose J, Klosterman SJ, Deelstra HJ, Ortiz-Castellanos L, Li W, Sanchez-Alonso P, Schreier PH, Häuser-Hahn I, Vaupel M, Koopmann E, Friedrich G, Voss H, Schlüter T, Margolis J, Platt D, Swimmer C, Gnirke A, Chen F, Vysotskaia V, Mannhaupt G, Güldener U, Münsterkötter M, Haase D, Oesterheld M, Mewes H-W, Mauceli EW, DeCaprio D, Wade CM, Butler J, Young S, Jaffe DB, Calvo S, Nusbaum C, Galagan J, Birren BW (2006) Insights from the genome of the biotrophic fungal plant pathogen *Ustilago maydis*. Nature 444:97–101 10.1038/nature05248 17080091

[CR14] Kim M, Jeong DW, Oh JW, Jeong HJ, Ko YJ, Park SE, Han SO (2022). Efficient synthesis of food-derived antioxidant l-ergothioneine by engineered *Corynebacterium glutamicum*. J Agric Food Chem.

[CR15] Kimura C, Nukina M, Igarashi K, Sugawara Y (2005). β-Hydroxyergothioneine, a new ergothioneine derivative from the mushroom *Lyophyllum Connatum*, and its protective activity against carbon tetrachloride-induced injury in primary culture hepatocytes. Biosci Biotechnol Biochem.

[CR16] Kitamoto H (2019). The phylloplane yeast *pseudozyma*: a rich potential for biotechnology. FEMS Yeast Res.

[CR17] Konishi M, Morita T, Fukuoka T, Imura T, Kakugawa K, Kitamoto D (2007). Production of different types of mannosylerythritol lipids as biosurfactants by the newly isolated yeast strains belonging to the genus *Pseudozyma*. Appl Microbiol Biotechnol.

[CR38] Liang C-H, Huang L-Y, Ho K-J, Lin S-Y, Mau J-L (2013) Submerged cultivation of mycelium with high ergothioneine content from the culinary-medicinal king oyster mushroom *Pleurotus eryngii* (higher Basidiomycetes) and its composition. Int J Medic Mushr 15:153–164 10.1615/intjmedmushr.v15.i2.40 23557367

[CR18] Lin S-Y, Chien S-C, Wang S-Y, Mau J-L (2015). Submerged cultivation of mycelium with high ergothioneine content from the culinary-medicinal golden oyster mushroom, *Pleurotus Citrinopileatus* (higher basidiomycetes). Int J Medic Mushr.

[CR19] Morita T, Takashima M, Fukuoka T, Konishi M, Imura T, Kitamoto D (2010). Isolation of basidiomycetous yeast *Pseudozyma tsukubaensis* and production of glycolipid biosurfactant, a diastereomer type of mannosylerythritol lipid-B. Appl Microbiol Biotechnol.

[CR20] Morita T, Ogura Y, Takashima M, Hirose N, Fukuoka T, Imura T, Kondo Y, Kitamoto D (2011). Isolation of *pseudozyma churashimaensis* sp. nov., a novel ustilaginomycetous yeast species as a producer of glycolipid biosurfactants, mannosylerythritol lipids. J Biosci Bioeng.

[CR21] Morita T, Koike H, Hagiwara H, Ito E, Machida M, Sato S, Habe H, Kitamoto D (2014). Genome and transcriptome analysis of the basidiomycetous yeast *Pseudozyma Antarctica* producing extracellular glycolipids, mannnosylerythritol lipids. PLoS ONE.

[CR22] Morita T, Fukuoka T, Imura T, Kitamoto D (2015). Mannosylerythritol lipids: production and applications. J Oleo Sci.

[CR23] Olicón-Hernández D, Araiza-Villanueva M, Pardo JP, Aranda E, Guerra-Sánchez G (2019). New insights of *Ustilago maydis* as yeast model for genetic and biotechnological research: a review. Curr Microbiol.

[CR26] Pfeiffer C, Bauer T, Surek B, Schomig E, Grundemann D (2011). Cyanobacteria produce high levels of ergothioneine. Food Chem.

[CR25] Pluskal T, Ueno M, Yanagida M (2014). Genetic and metabolomic dissection of the ergothioneine and selenoneine biosynthetic pathway in the fission yeast, S. Pombe, and construction of an overproduction system. PLoS ONE.

[CR27] Saika A, Koike H, Yamamoto S, Kishimoto T, Morita T (2017). Enhanced production of a diastereomer type of mannosylerythritol lipid-B by the basidiomycetous yeast *Pseudozyma tsukubaensis* expressing lipase genes from *Pseudozyma Antarctica*. Appl Microbiol Biotechnol.

[CR28] Saika A, Fukuoka T, koike H, Yamamot S, Sugahara T, Sogabe A, Kitamoto D, Morita T (2020). A putative transporter gene PtMMF1-deleted strain produces mono-acylated mannosylerythritol lipids in *Pseudozyma tsukubaensis*. Appl Microbiol Biotechnol.

[CR29] Servillo L, Castaldo D, Casale R, D’Onofrio N, Giovane A, Cautela D, Balestrieri ML (2015). An uncommon redox behavior shed light on the cellular antioxidant properties of ergothioneine. Free Rad Biol Med.

[CR30] Smith E, Ottosson F, Hellstrand S, Ericson U, Orho-Melander M, Fernandez C, Melander O (2020). Ergothioneine is associated with reduced mortality and decreased risk of cardiovascular disease. Heart.

[CR31] Stoffels C, Oumari M, Perrou A, Termath A, Schlundt W, Schmalz H-G, Schafer M, Wewer V, Metzger S, Schomig E, Grundemann D (2017). Ergothioneine stands out from hercynine in the reaction with singlet oxygen: resistance to glutathione and TRIS in the generation of specific products indicates high reactivity. Free Rad Biol Med.

[CR32] Takusagawa S, Satoh Y, Ohtsu I, Dairi T (2019). Ergothioneine production with *aspergillus oryzae*. Biosci Biotechnol Biochem.

[CR33] Tanaka N, Kawano Y, Satoh Y, Dairi T, Ohtsu I (2019). Gram-scale fermentative production of ergothioneine drive by overproduction of cysteine in *Escherichia coli*. Sci Rep.

[CR39] Tepwong P, Giri A, Sasaki F, Fukui R, Ohshima T (2012) Mycobial enhancement of ergothioneine by submerged cultivation of edible mushroom mycelia and its application as an antioxidative compound. Food Chem 131:247–258

[CR9] van der Hoek SA, Darbani B, Zugaj KE, Prabhala BK, Biron MB, Randelovic M, Medina JB, Kell DB, Borodina I (2019). Engineering the yeast *Saccharomyces cerevisiae* for the production of l-(+)-ergothioneine. Front Bioeng Biotechnol.

[CR10] van der Hoek SA, Rusnak M, Jacobsen IH, Martinez JL, Kell DB, Borodina I (2022). Engineering ergothioneine production in *Yarrowia Lipolytica*. FEBS Lett.

[CR11] van der Hoek SA, Rusnak M, Wang G, Stanchev LD, Alves LF, Jessop-Fabre MM, Paramasivan K, Jacobsen IH, Sonnenschein N, Martinez JL, Darbani B, Kell DB, Borodina I (2022). Engineering precursor supply for the high-level production of ergothioneine in *Saccharomyces cerevisiae*. Metab Eng.

[CR35] Wada K, Koike H, Morita T (2021). Draft genome sequence of a basidiomycetous yeast, *Ustilago Shanxiensis* CBS10075, which produces mannoyslerythritol lipids. Microbiol Resour Announc.

[CR34] Yang N-C, Lin H-C, Wu J-H, Ou H-C, Chai Y-C, Tseng C-Y, Liao J-W, Song T-Y (2012). Ergothioneine protects against neuronal injury induced by β-amyloid in mice. Food Chem Toxicol.

[CR36] Zhang L, Tang J, Feng M, Chen S (2023). Engineering methyltransferase and sulfoxide synthase for high-yield production of ergothioneine. J Agric Food Chem.

[CR37] Zhu M, Han Y, Hu X, Gong C, Ren L (2022). Ergothioneine production by submerged fermentation of a medicinal mushroom *Panus conchatus*. Fermentation.

