# Supplementary material for: Biosynthetic ability of diverse basidiomycetous yeast strains to produce the natural antioxidant ergothioneine
Source: AMB Express. 2024 Feb 9;14:20. doi: 10.1186/s13568-024-01672-w (PMC10858013; doi:10.1186/s13568-024-01672-w)
Supplement: Supplementary file 1 — Supplementary Material 1: Fig. S1: Effect of culture conditions on EGT production by (A) U. siamensis, (B) U. shanxiensis, and (C) M. antarcticus [file 13568_2024_1672_MOESM1_ESM.docx]

*AMB Express*

**Biosynthetic ability of diverse basidiomycetous yeast strains to produce the natural antioxidant ergothioneine**

Shun Sato^1^, Azusa Saika^1^, Kazunori Ushimaru^1^, Tatsuyuki Koshiyama^2^, Yukihiro Higashiyama^2^, Tokuma Fukuoka^1^, Tomotake Morita^1^*

^1^ Research Institute for Sustainable Chemistry, National Institute of Advanced Industrial Science and Technology (AIST), Central 5-2, 1-1-1 Higashi, Tsukuba, Ibaraki 305-8565, Japan

^2^ Research and Development Division, Kureha Corporation, 16, Ochiai, Nishiki-Machi, Iwaki, Fukushima 974-8686, Japan

* Correspondence to: Tomotake Morita, E-mail, morita-tomotake@aist.go.jp; Tel, +81-29-861-4426

Fig. S1. Effect of culture conditions on EGT production by (A) *U. siamensis*, (B) *U. shanxiensis*, and (C) *M. antarcticus*.

Cells were cultivated in yeast mold (YM) medium modified with indicated conditions for 5 days (n=1). EGT inside cells was measured by liquid chromatography-mass spectrometry (LC-MS) as described in Materials and Methods. Dotted lines show the EGT production level in YM medium (containing 10 g/L glucose, 5 g/L peptone, 3 g/L yeast extract, and 3 g/L malt extract) at 25^o^C and 200 rpm for 120 h by each strain as the standard condition. Final concentrations of glucose, yeast extract, and peptone were shown. Salinity was modified with the addition of NaCl. Initial pH of YM medium was adjusted by 1 M HCl or 1 M NaOH. Tr, trace (less than 1 mg/L)
